# Construction practice of water conveyance tunnel among complex geotechnical conditions: a case study

**DOI:** 10.1038/s41598-023-42192-0

**Published:** 2023-09-12

**Authors:** Kaimin Duan, Guofeng Zhang, Hui Sun

**Affiliations:** 1 Changjiang Institute of Technology, Wuhan, Hubei China; 2 China South-to-North Water Diversion Jianghan Water Network Construction and Development Co. Ltd, Wuhan, Hubei China; 3 School of Civil Engineering, Henan Polytechnic University, Jiaozuo, Henan China

**Keywords:** Ecology, Environmental sciences, Hydrology, Natural hazards

## Abstract

The construction practice of water conveyance tunnels often encounters various complex geotechnical engineering conditions, which bring huge challenges to the design and construction of water conveyance tunnels. Based on the theory of rock elastic–plastic mechanics and finite element analysis technology, this article carried out investigations of engineering geological features, geological formations and hydrological conditions establishes a calculation model for the 3# water conveyance tunnel of the Fenhe River Diversion Project, and analyzes the variation law of surrounding rock stress and displacement during TBM excavation of the tunnel. The results indicate that the dominant direction of the rock mass principal stress measured by the hydraulic fracturing method is NE84°, and the maximum horizontal principal stress, minimum horizontal principal stress, and vertical stress decrease sequentially, analyzing the characteristics of shield TBM construction technology, it is applied to the construction of water transfer tunnels. The numerical simulation of TBM construction using FLAC3D software shows that as the excavation surface advances, the subsidence value of the tunnel roof first slowly increases, then rapidly increases, and then tends to stabilize. The horizontal displacement of the surrounding rock is increasing. The maximum principal stress of the surrounding rock gradually increases. The final surrounding rock stress is 35 MPa. The TBM shield machine with mud water balance driven by indirectly controlled frequency conversion motor is selected for TBM construction of the tunnel. The study offers statistical information to support tunneling technology for water conveyance in the geotechnical engineering practice.

## Introduction

With the rapid development of cities in China and the deepening of urbanization, the scale, population and volume of cities are constantly increasing, the demand for water is also increasing, and the problem of urban water shortage is becoming more and more serious^1^. Although China has abundant water resources, the distribution of water resources in various regions is relatively uneven, and the arid/semi-arid land area accounts for about 52%. There are about 450 large and medium-sized cities with water shortage. At present, the main sources of urban domestic water are groundwater and surface water. Overexploitation of groundwater in cities has led to the decline of groundwater level, resulting in a large number of engineering problems such as large-scale ground subsidence. The state has promoted the search and construction of new water source projects. Many cross regional water diversion projects are constantly emerging in various cities, and at the same time, the construction of a quantity of water tunnels has also followed. When the water conveyance tunnel is being built, various complicated geological conditions are often encountered, which puts forward higher requirements for tunnel construction technology and brings great challenges to construction safety and construction progress^2^. Therefore, it is of great significance to study the construction technology of water conveyance tunnel under complex geological conditions.

In the large-scale trans-regional water diversion project, the water conveyance tunnel is the most important main project in the whole project construction, and it is also the most critical factor affecting the whole project progress^3^. Water tunnels often encounter various types of complex geological problems. Failure to solve the complex geological problems can lead to various tunnel diseases and cause unforeseen damages to the project. In order to prevent and manage the tunnel damage and to ensure the safety and stability of the tunnel during the construction and operation phases, it is necessary to know the common tunnel damage and the complex geostress situation. According to the complex geological problems that may occur in water transfer tunnels, from the perspective of construction risk, they can be mainly divided into hard rock rock bursts, large soft rock deformation, water and mud surges, fracture zones, toxic and hazardous gases, high ground temperatures and radioactive substances, and so on. With the construction of more and more water diversion projects and water conservancy projects, a variety of more complex problems have gradually surfaced, such as the high geopathic stress bow} water tunnels buried deep in the ground, where the surrounding rock environment is complex, and the lining and the surrounding rock synergistic effect after tunnel excavation and the mechanism of destruction of the two is also intricate and complex. Although many practical technical problems have been solved in a large number of cases in recent years, there are still a large number of outstanding technical and theoretical problems to be solved.

Some researchers have carried out the research on the main geological problems faced by the water conveyance tunnel and the tunnel size setting, and summarized the situations such as rock burst, deformation of surrounding rock, water inrush, fault zone, toxic gas and high ground temperature that are often encountered in tunnel construction^4–6^. At the same time, the tunnel's diameter, length, and depth must also be thoughtfully planned while taking into account the route's complicated and shifting geological circumstances^7^. Some researchers have carried out tunnel construction research under the harsh geological conditions of high stress and high permeability^8–10^. Due to the restriction of terrain and excavation equipment, the engineering difficulty of survey and design is increased, and the construction progress is slow. Based on rock mechanics and support theory, advanced bolting and shotcreting support technology is used to establish new Austrian tunneling method (NATM), shallow buried excavation method and other construction methods which should be in different geological conditions^11^. Some researchers put forward the construction method of drilling and blasting with hydraulic rock drill^12^. Drilling holes to carry out blasting excavation, blasting surrounding rocks with explosives, which caused large-scale disturbance and damage of surrounding rocks, is widely used in metamorphic rocks and igneous rocks^13^. At the same time, with the continuous improvement of blasting technology and level, the blasting range is controlled by adjusting the number of explosives, and drilling and blasting construction technology is also well used in soft rocks or hard soils^14^. Full-face tunnel boring machine (TBM) have been proposed by several researchers as a method of tunnel excavation^15^. Based on large-scale modern transportation mechanization, TBM is used as the main equipment, and the mechanical pressure means is used to complete the cutting, crushing, excavation, transportation, lining and grouting of rock at one time, which greatly improves the construction progress, has good construction safety, low requirements for ventilation conditions and less supporting work^16^. At the same time, according to different geological conditions, technical means such as single shield, double shield and three shield are proposed to adapt to various geological environments and geological bodies^10^. Some researchers have studied the influence of different geological and topographical conditions on tunnel construction^17^. Different tunnel construction methods have been suggested for a variety of complex geological structures based on the actual engineering situation. These methods include shallow buried asymmetric tunnels, deep buried tunnels in weak surrounding rocks, landslide geological body tunnels, etc., which can effectively prevent water seepage and support excavations while also protecting the environment and construction safety^18^. Based on the control of tunnel construction progress, some researchers put forward the research ideas, technical routes and construction methods of underground tunnel-group construction, developed the online monitoring system of underground tunnel-group construction progress, and applied it to hydropower stations, water diversion and other large-scale water conservancy projects, and achieved good results in improving the construction progress^19^. Some researchers have carried out the comparison between the traditional drilling and blasting method and the full-face tunneling method, and concluded that the tunneling speed of TBM is much higher than that of drilling and blasting method. At the same time, TBM construction technology is more environmentally friendly and labor-saving, which helps to improve the construction efficiency and economic benefits^20^. Some researchers have summarized the advantages and disadvantages of TBM construction technology^21^. The main advantages are: continuous construction, integration of construction procedures, small disturbance and damage to surrounding rock, smooth excavation surface, relatively small vibration and noise, reduced labor cost, good working environment and improved construction safety. However, there are also some disadvantages, such as complicated supporting system construction, large investment in the early stage, single construction mode, high requirements for operators and geological conditions. Some researchers have made statistics on the construction technology of main water conveyance tunnels in China at present^22^. Table 1 shows the statistical results. TBM technology has become the main technology of tunnel construction.

**Table 1:** Statistics of tunnel construction technology of major water conservancy projects in China.

Time	Project	Excavation diameter (M)	Tunnel length (M)	Construction technology
2011	Tianshengqiao Hydropower Station Project	10.8	10	TBM
2015	Gansu Datong River District Diversion Project	5.5	11.7	TBM
2018	Shanxi Yellow River Diversion Project	5.6	123	TBM

With the continuous improvement of China's urbanization, the demand for water resources in major cities is increasing rapidly. Building large-scale water conservancy projects such as water diversion, water delivery and water supply has become the main solution to water shortage. In this paper, taking Fenhe Water Diversion Project in Shanxi Province as the research object, the on-site geological survey was carried out, and the geological characteristics, geological structure and hydrological conditions of the project were analyzed. Aiming at the influence of complex geological conditions on tunnel construction, based on the theory of rock elastic–plastic mechanics and finite element analysis technology, the calculation model of Fenhe Water Diversion Project 3# water conveyance tunnel was established, and the variation law of surrounding rock stress and displacement during tunnel TBM excavation was analyzed. Shield TBM construction technology was applied to water conveyance tunnel construction, providing theoretical data support for the construction technology of water conveyance tunnel in large-scale water conservancy projects.

## Geological and geotechnical setting

Water conveyance tunnel construction is often faced with different types of complex geological conditions. If these geological problems are not solved, it will have an important impact on the tunnel, and may also produce different hazards and unforeseen accidents. In order to effectively prevent engineering accidents and ensure the safety of tunnel construction and operation, different construction measures are established by analyzing the mechanism of tunnel diseases caused by different geological conditions. With the construction of large-scale water diversion and water conservancy projects, complex geological problems emerge in endlessly, such as the common construction problems of deep underground tunnels with high geostress tunnels. New technologies and theories need to be further improved to solve these complex geological problems.

### Geological characteristics

The Fenhe River Water Diversion Project in Shanxi Province is an important water conservancy project to solve the problem of water use in Shanxi Province. The project starts from the Tianqiao Hydropower Station, passes through five cities and sixteen counties, and reaches Taiyuan City, mainly including water intake, water conveyance and water diversion projects. The project passes through the Luliang Mountains and connects to the tributaries of the Yellow River and the Fenhe River system, mainly including Zhujia River, Yifen River, Sanchuan River and other first-class tributaries of the Yellow River, and Fenhe River tributaries such as the Beichuan River, Dongchuan River and Nanchuan River^23^.

The water diversion project includes the main trunk line, the east and west branch lines and various water delivery points. The project spans the western Loess Plateau and Luliang Mountain fault area and extends from north to south. The landforms along the project include loess hilly area, Luliang Mountain fold fault area, broken loess area, arch erosion platform area, etc. Quaternary clay layer and Tertiary low liquid limit clay layer are mainly distributed along the surface of the project, and the bedrock strata are Archean sedimentary rocks, Carboniferous, Ordovician, Permian and Mesozoic Triassic from old to new.

The structural features of the project include folds and large faults from south to north, such as Lishi large fault, Haojiagou anticline, Dawuzhike syncline, Nuanquan anticline, Lishi syncline and Luocun Baiguishan fold. Through the value of seismic response period along the line, the seismic intensity of the section where the project is located is zero. The geological features of the project area include landslide, bedrock weathering and collapse. Different stratigraphic combinations and structural conditions lead to great differences in the weathering degree of rock mass. Carbonate rocks and caves and holes of different sizes are mainly exposed along the way, and no landslide is developed. No landslide body is found, but after the geological conditions change, a small-scale collapse will occur.

Groundwater types along the water diversion tunnel mainly include fissure water, fissure karst water and interlayer water. The karst water in carbonate rock and the fissure water in metamorphic rock have a great impact on the tunnel construction. Groundwater mainly exists in Carboniferous sandstone and limestone, and aquifers are developed in shale and mudstone. The groundwater is mainly supplied by atmospheric precipitation and pore water. The water quality is average, even worse near the coal seam.

When monitoring in tunnels, commonly used instrumentation includes: inclinometers, settlement meters, tensiometers, strain gauges, steel strain gauges, manometers, etc. Among them, settlement meters: are used to measure the settlement of tunnel roofs, foundations and supporting structures, strain gauges: are used to measure the strains in the tunnel structure, and manometers: are used to measure the water pressures or geopathic stresses in the groundwater or the rock mass. Table 2 shows Physical and mechanical parameters of surrounding rock.

**Table 2:** Physical and mechanical parameters of surrounding rock.

Depth (m)	Capacity (kN/m^3^)	Modulus of elasticityE (GPa)	Cohesion (MPa)	Friction angle (°)	Poisson	Lateral pressure coefficient K_x_	Lateral pressure coefficient tK*y*
450	25.8	7.0	0.78	36	0.32	1.42	1.05

### Geostress field and rock mechanical properties

In this paper, the rock mass of 3# conveyance tunnel of water diversion project is selected as the research object, and the tunnel is drilled with a depth of about 300 m, and the geostress of the rock mass is measured by hydraulic fracturing method. Table 3 shows the test results of borehole geostress. It can be seen that the dominant direction of the main stress in the rock mass is NE84°, which is basically parallel to the axis of the water diversion tunnel and has no impact on the surrounding rock of the water diversion tunnel. The maximum horizontal principal stress, the minimum horizontal principal stress and the vertical stress decrease successively, so the crustal stress is dominated by tectonic stress. Although the geostress is relatively high, it is all less than 20 MPa, which is not very high. Along the water diversion project, the buried depth of the deepest tunnel is 650 m, but the maximum depth of the test borehole is only 500 m at present. Therefore, this paper uses the linear regression method to estimate the geostress of rock masses with different depths^24^. Linear regression calculations of the borehole stress values yielded the following expression for the linear regression relationship:

1 $${\text{Maximum }}\;{\text{horizontal}}\;{\text{ principal }}\;{\text{stress }}\;{\text{value}}\, = \,0.0{\text{269H}}\, + \,{4}.{3112}$$

**Table 3:** Geostress results of hydraulic fracture in borehole.

Drilling depth (m)	Maximum horizontal principal stress value (MPa)	Minimum horizontal principal stress value (MPa)	Vertical stress value (MPa)	Fracture direction (°)
140	7.11	4.73	3.51	
145	8.44	6.11	3.82	NE82°
153	9.18	6.71	3.99	
180	9.31	7.57	4.26	NE86°
195	9.76	7.54	5.21	
210	10.01	7.01	5.49	
253	10.23	7.18	6.23	
300	12.78	8.32	7.33	

**Figure 1:**
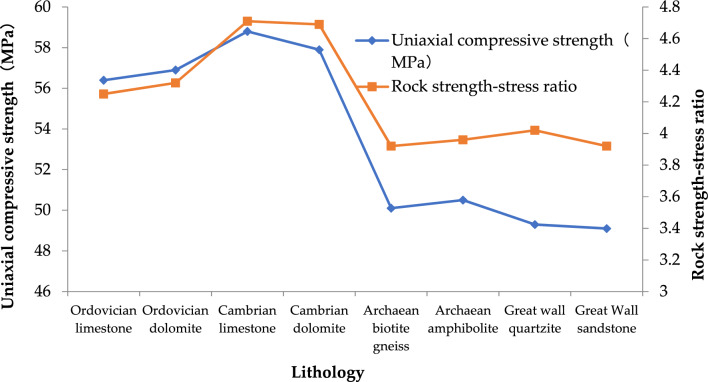
Uniaxial compressive strength of rocks with different lithology.

Ordovician and Cambrian dolomite and limestone, Archaean biotite gneiss, amphibolite and gneiss, Great Wall quartzite and sandstone are mainly distributed along the water conveyance tunnel. In this study, rock-related physical and mechanical investigations are used to determine the uniaxial compressive strength (Table 4). Figure 1 shows the uniaxial compressive strength and rock strength-stress ratio of rocks with different lithology. It can be seen from the figure that the uniaxial compressive strength of Ordovician rocks is 56.65 MPa, that of Cambrian rocks is 58.35 MPa, that of Archaeozoic rocks is 50.30 MPa, that of Great Wall rocks is 49.2 MPa, and that of Cambrian rocks is the largest on the whole. The strength-stress ratio of Ordovician rocks is 4.285, that of Cambrian rocks is 4.70, that of Archaeozoic rocks is 3.94, and that of Great Wall rocks is 3.97. On the whole, the strength-stress ratio of Ordovician rocks is the highest.

**Table 4:** Uniaxial compressive strength of rocks with different lithology.

Lithology	Uniaxial compressive strength (MPa)	Rock strength-stress ratio
Ordovician limestone	56.4	4.25
Ordovician dolomite	56.9	4.32
Cambrian limestone	58.8	4.71
Cambrian dolomite	57.9	4.69
Archaean biotite gneiss	50.1	3.92
Archaean amphibolite	50.5	3.96
Great wall quartzite	49.3	4.02
Great Wall sandstone	49.1	3.92

## Numerical modeling results

In this paper, numerical simulation and analysis are carried out on the excavation construction of 3# water conveyance tunnel of water diversion project. The tunnel adopts TBM full face excavation method, and the initial geostress field is set as gravity. Based on these considerations, this paper simulates the synchronous excavation and support situation under high water pressure geological settings, examines the tunneling process, and assesses changes in stress and displacement of the surrounding rock.

In the numerical simulation analysis, the rock mass is regarded as homogeneous and isotropic elastic–plastic material, and the deformation time of rock mass and groundwater seepage are ignored. The numerical calculation model consists of an axial length of 30 m on both sides of the tunnel, a transverse length of 60 m, and a longitudinal length of 30 m, totaling 60 m. The size of the model is 48 m × 60 m × 60 m. In this paper, FLAC3D software is used for simulation. Fixed constraints are used on the model floor, normal displacement constraints are used on the four sides, the self-weight of the overlying surrounding rock is considered on the top surface, and the initial stress field is loaded in the overall model.

**Figure 2:**
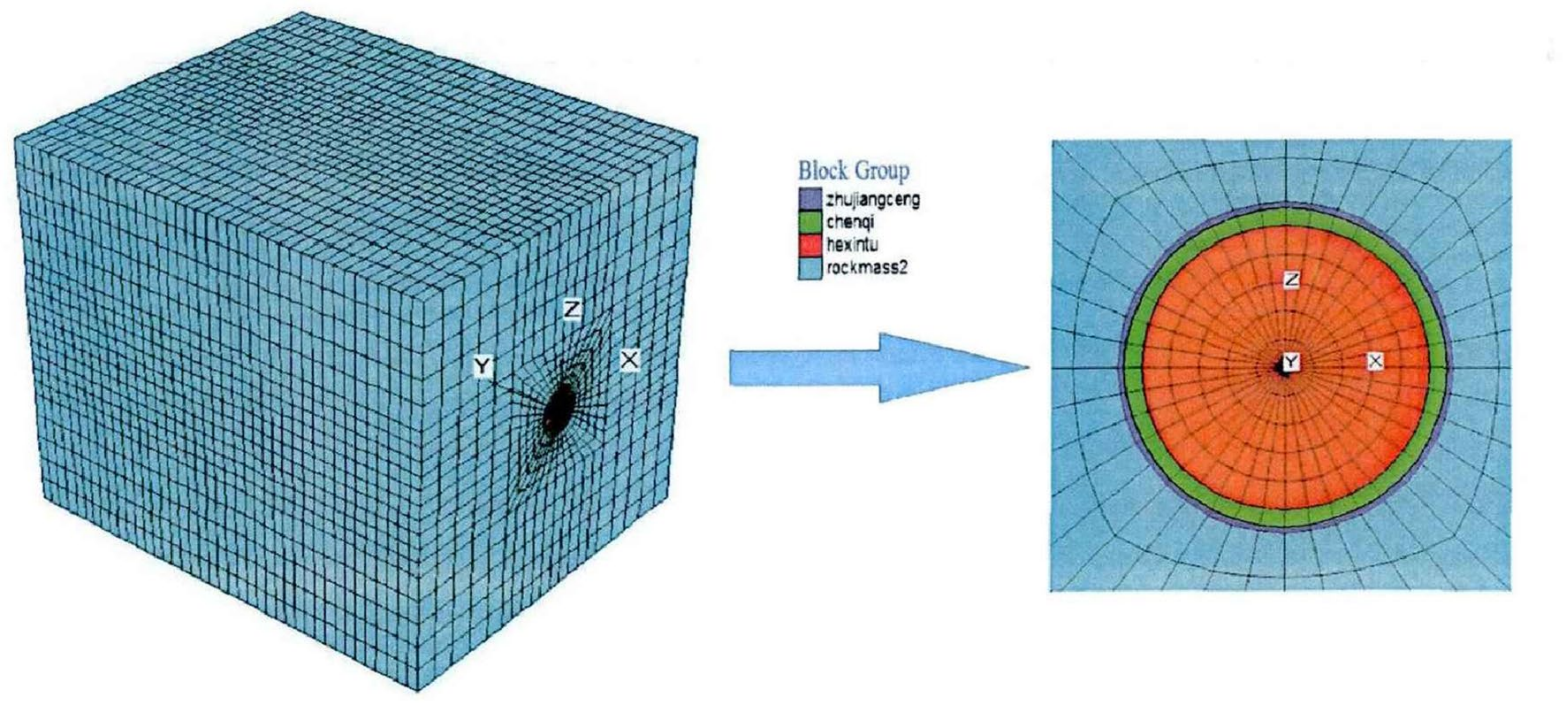
FLAC3D numerical calculation model.

When using FLAC3D for numerical calculations, the smaller the grid, the better the calculation results, but the number of grids is limited by the program and computer hardware, and also too many grids will take more calculation time. The shape of the calculation grid also has a significant effect on the calculation results. Therefore, according to the actual engineering examples and the limitations of computer hardware, the model grid in this paper adopts the hexahedral cell dissection grid, which has 31,200 cells and 32,841 nodes. The numerical calculation model of FLAC3D is shown in Fig. 2.

The excavation was supported immediately after excavation under high external water pressure, with 1.2 m as one excavation step. Each excavation step is divided into two time steps, the first time step simulates tunnel excavation, applying top thrust and grouting pressure on the palm face; the second time step simulates pipe sheet installation.

When simulating tunnel excavation, the stress and deformation of the surrounding rock will change due to the excavation surface effect. The tunnel section is taken as the monitoring surface, and Table 5 shows the influence of excavation face advancing on the displacement of surrounding rock at the roof of the tunnel.

**Table 5:** Influence of excavation face advancing on the displacement of surrounding rock at the roof of the tunnel.

Distance to monitoring section L	Roof subsidence value (cm)	Horizontal displacement (cm)	Displacement release coefficient (%)
-D	0.002	0	0.1
− 0.5D	0.01	0	2.5
0D	0.32	0	13.4
0.5D	2.04	0.1	88.3
1D	2.15	0.5	93.2
2D	2.52	2.5	99.3
3D	2.53	2.6	100
4D	2.53	2.6	100

**Figure 3:**
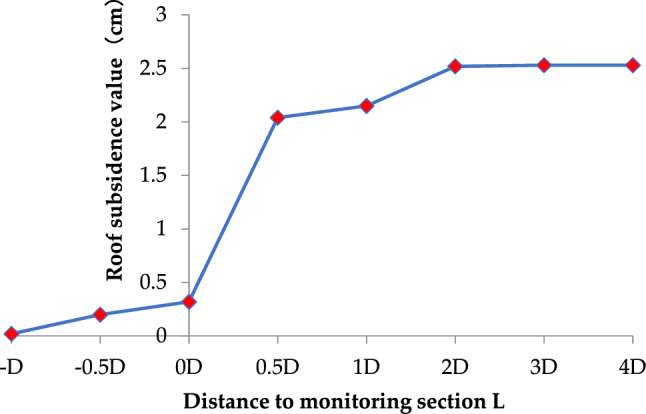
The roof subsidence value of the tunnel with the excavation face advancing.

Figure 3 shows the roof subsidence value with the excavation face advancing. It can be seen from the figure: with the excavation face advancing, the roof subsidence value increases slowly at first, then quickly, and then tends to be stable. When the excavation face is 0.5 times the tunnel diameter from the monitoring face, the roof displacement is 88.3%. When the distance between the excavation face and the monitoring end face is twice the tunnel diameter, the displacement of the tunnel roof is 99.3%. It can be considered that the excavation face effect disappears when the distance between the excavation face and the monitoring face exceeds 1–2 times the tunnel diameter. Analyses are being conducted at the same time to determine how the excavation face affects the tunnel's horizontal displacement. With the excavation face advancing, the horizontal displacement of the surrounding rock increases continuously. The horizontal movement of the surrounding rock grows to 0.5 cm at a distance of one time the tunnel's diameter between the excavation surface and the monitoring surface. The horizontal movement of the surrounding rock quickly rises to 2.5 cm, and the displacement release coefficient reaches 99%, which is consistent with the vertical deformation law of the surrounding rock, as the space between the excavated surface and the monitoring surface is twice the tunnel's diameter.

**Figure 4:**
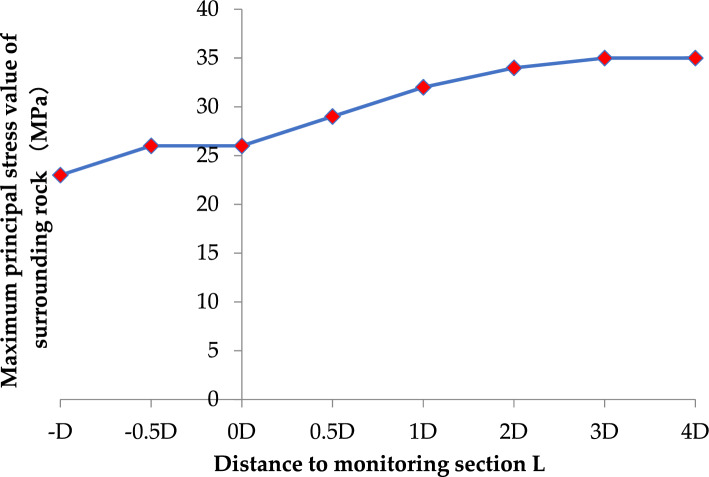
Relationship between the distance of excavation face and end face and the maximum principal stress of surrounding rock.

Table 6 shows the change of the maximum principal stress of surrounding rock with the excavation face advancing. It can be seen from Table 5 that with the increase of the distance between the excavation face and the end face, the maximum principal stress of the surrounding rock gradually increases, and finally tends to be stable. When the distance between the excavation face and the end face is twice the diameter of the tunnel, the surrounding rock stress increases by 11 MPa, and then with the increase of the distance between the excavation face and the end face, the surrounding rock stress area is stable, and finally reaches 35 MPa. Figure 4 shows the relationship between the distance of the excavation face and the end face and the maximum principal stress of surrounding rock. In the process of advancing the excavation face, the segment stress slowly advances along with the excavation face, and finally stabilizes at about 32 MPa.

**Table 6:** Maximum principal stress change of surrounding rock with the excavation face advancing.

Distance to section	− D	− 0.5D	0D	0.5D	1D	2D	3D	4D
Maximum principal stress value of surrounding rock (MPa)	− 23	− 26	− 28	− 29	− 32	− 34	− 35	− 35

The calculation results of FLAC3D model show that the sensitivity of each parameter to the stability of the surrounding rock, among which the angle of internal friction of the surrounding rock has the most significant influence on the stability of the surrounding rock. In the actual engineering numerical calculation, the value of the surrounding rock parameters should be paid enough attention to provide a reliable basis for future design and construction.

## Discussion

In this paper, TBM construction is adopted for the 3# water conveyance tunnel of the water diversion project. The construction support design is required, and the main work of the initial support is as follows. Pay attention to equipment safety while ensuring surrounding rock support; the support speed shall be synchronized with the excavation speed, and the slow support shall not affect the project excavation progress; the support shall be in place at one time, without secondary supplement, and advance support shall be provided; reduce support workload, improve work efficiency and save construction cost under the condition of ensuring safety.

The key of TBM construction is the selection of TBM machine. Considering the reliability of the equipment and the economy of the project, auxiliary construction and redundant processes should be reduced as much as possible when selecting the tunnel boring machine. At the same time, the equipment selection needs to be based on the geological conditions of the construction area, the tunnel size^25^.

This study selected earth pressure balanced TBM shield and mud water balanced TBM shield, based on the water level situation of the Fenhe River Diversion Project in Shanxi Province. For tunnels with sandstone, glutenite and mud conglomerate, TBM shield equipment with balanced earth pressure is generally used, and additives need to be added in the construction process to improve and enhance the stability of ballast soil. When gravel and mud conglomerate are excavated, it is found that there is a high degree of breakage and there is a large granular ballast soil, the screw conveyor is used to judge the particle size. For tunnels in silty soil, sand and gravel layers, TBM shield with mud-water balance is generally used, and a crusher is built in the balance bin^26^.

The hydraulic conductivity is also the main factor affecting equipment selection^27^. When the hydraulic conductivity of rock mass is less than 10^−7^ cm/s, TBM shield with earth pressure balance is generally selected. When the hydraulic conductivity of rock mass is between 1^−7^ and 10^−4^ cm/s, TBM shield with mud-earth pressure balance and TBM shield with mud-water balance can be selected. When the hydraulic conductivity of rock mass is greater than 10^−4^ cm/s, in view of the increase of construction difficulty, TBM shield with mud-water balance is generally used. The hydraulic conductivity of rock mass of 3# water conveyance tunnel in Shanxi Fenhe Water Diversion Project exceeds 10^−4^ cm/s, so TBM shield with mud-water balance is used.

The driving modes of TBM mainly include frequency conversion, hydraulic pressure and fixed speed. Because the rotating speed of cutter head driven by constant speed motor cannot be adjusted, it is generally not used in engineering construction. Table 7 shows the comparison between frequency conversion and hydraulic drive^28^. Through comprehensive comparison, TBM driven by frequency conversion motor is adopted in the construction of 3# water conveyance tunnel.

**Table 7:** Comparison between frequency conversion and hydraulic drive.

Project	Frequency conversion	Hydraulic drive
Overall dimension of driving part	Big	Small
Subsequent equipment	Less	More
Efficiency (%)	95	65
Starting current	Small	Small
Starting torque	Big	Small
Starting impact	Small	Less
Speed control and fine adjustment	Good	Good
Noise	Low	High
Maintaining	Easy	Difficult

Mud-water balancing TBM shields can be grouped into direct control and indirect control depending on the structural makeup of the balance bin of the TBM construction machine and the slurry pressure control mode. The mud-water pressure of the mud-water shield is indirectly controlled by the air pressure mode, and the slurry support pressure of the surrounding rock of the excavation face is established, on the basis of properly adjusting the air pressure, so the indirect control mud-water balance TBM shield is selected.

TBM construction procedure is as follows.

1. The TBM construction machine starts the foundation reinforcement in the construction section.
2. The shaft of TBM shield should be installed in place in advance.
3. The tunneling face of TBM shield is broken first, and the shaft is arranged from north to south.
4. TBM shield tunneling forward while continuously installing lining segments.
5. After TBM shield tunneling to the construction shaft, repair and maintain the equipment, transport the equipment of the previous mud-water treatment system to the south bank, start tunneling, and prefabricate the segments in advance.
6. Tunnels at both ends are excavated at the same time, and the surrounding rock of the tunnel is lined with secondary concrete.
7. When the TBM shield is 10 m away from the entrance of the tunnel, the shield tunneling construction is completed. After that, the excavation is done using the mining technique, and the surrounding rock is supported. And the patched bottom partial segment is adopted to excavate the earth and rock. Finally, push it out of the tunnel and dismantle it.
8. The tunnel is completed after the secondary concrete lining is carried out and the surrounding rock is well supported.

## Conclusions

The construction practice of water conveyance tunnels often encounters various complex geotechnical engineering conditions, which bring huge challenges to the design and construction of water conveyance tunnels. Taking the Fenhe River Water Diversion Project in Shanxi Province as the research object, this paper investigates the engineering geological characteristics, tectonics and hydrological conditions, analyzes the influence of complex geological conditions on tunnel construction, and puts forward effective construction technical measures. The main research results are as follows.

1. The geostress of rock mass is measured by hydraulic fracturing method. The dominant direction of rock mass in water conveyance tunnel is NE84°, with the maximum horizontal principal stress, the minimum horizontal principal stress, and the vertical stress decreasing sequentially.
2. The numerical simulation of TBM construction was carried out by FLAC3D software, and it was found that with the advance of the excavation face, the roof subsidence value increased slowly at first and then quickly, and then tended to be stable. When the distance between the excavation face and the monitoring face was twice the diameter of the tunnel, the roof displacement was 99.3%. With the advance of the excavation face, the horizontal displacement of the surrounding rock increases continuously. The horizontal movement of the surrounding rock quickly rises to 2.5 cm, and the displacement release coefficient reaches 99%, which is consistent with the vertical deformation law of the surrounding rock, as the space between the excavated surface and the monitoring surface is twice the tunnel's diameter. With the distance between excavation face and end face increasing, the maximum principal stress of surrounding rock gradually increases, and the final stress of surrounding rock is 35 MPa. The TBM construction support design was carried out, and the TBM shield machine with mud-water balance driven by an indirect control motor with frequency conversion was selected to establish the TBM construction procedure.

However, in the selection of long tunnel routes, due to the limitations of current exploration methods, geological exploration cannot be done in a very detailed manner, resulting in limitations in understanding geological structures. In order to ensure the normal construction of the tunneling machine and timely understand the geological conditions in front of the construction site, it is necessary to carry out accurate advanced geological exploration, prediction, and processing technology and related equipment research.

## Data Availability

The figures and tables used to support the findings of this study are included in the article.
